# Mechanical, Thermodynamic and Electronic Properties of Wurtzite and Zinc-Blende GaN Crystals

**DOI:** 10.3390/ma10121419

**Published:** 2017-12-12

**Authors:** Hongbo Qin, Xinghe Luan, Chuang Feng, Daoguo Yang, Guoqi Zhang

**Affiliations:** 1 School of Mechanical and Electronic Engineering, Guilin University of Electronic Technology, Guilin 541004, China; qinhb@guet.edu.cn (H.Q.); 1601201040@mails.guet.edu.cn (X.L.); 1601201020@mails.guet.edu.cn (C.F.); g.q.zhang@tudelft.nl (G.Z.); 2 EEMCS Faculty, Delft University of Technology, 2628 Delft, The Netherlands

**Keywords:** GaN, mechanical property, thermodynamic property, anisotropy, electronic property, first principle

## Abstract

For the limitation of experimental methods in crystal characterization, in this study, the mechanical, thermodynamic and electronic properties of wurtzite and zinc-blende GaN crystals were investigated by first-principles calculations based on density functional theory. Firstly, bulk moduli, shear moduli, elastic moduli and Poisson’s ratios of the two GaN polycrystals were calculated using Voigt and Hill approximations, and the results show wurtzite GaN has larger shear and elastic moduli and exhibits more obvious brittleness. Moreover, both wurtzite and zinc-blende GaN monocrystals present obvious mechanical anisotropic behavior. For wurtzite GaN monocrystal, the maximum and minimum elastic moduli are located at orientations [001] and <111>, respectively, while they are in the orientations <111> and <100> for zinc-blende GaN monocrystal, respectively. Compared to the elastic modulus, the shear moduli of the two GaN monocrystals have completely opposite direction dependences. However, different from elastic and shear moduli, the bulk moduli of the two monocrystals are nearly isotropic, especially for the zinc-blende GaN. Besides, in the wurtzite GaN, Poisson’s ratios at the planes containing [001] axis are anisotropic, and the maximum value is 0.31 which is located at the directions vertical to [001] axis. For zinc-blende GaN, Poisson’s ratios at planes (100) and (111) are isotropic, while the Poisson’s ratio at plane (110) exhibits dramatically anisotropic phenomenon. Additionally, the calculated Debye temperatures of wurtzite and zinc-blende GaN are 641.8 and 620.2 K, respectively. At 300 K, the calculated heat capacities of wurtzite and zinc-blende are 33.6 and 33.5 J mol^−1^ K^−1^, respectively. Finally, the band gap is located at the G point for the two crystals, and the band gaps of wurtzite and zinc-blende GaN are 3.62 eV and 3.06 eV, respectively. At the G point, the lowest energy of conduction band in the wurtzite GaN is larger, resulting in a wider band gap. Densities of states in the orbital hybridization between Ga and N atoms of wurtzite GaN are much higher, indicating more electrons participate in forming Ga-N ionic bonds in the wurtzite GaN.

## 1. Introduction

Group-III nitride semiconductors have attracted considerable attention in recent years because of their great potential for technological applications such as short-wavelength light-emitting diodes (LEDs), photocatalysts, optoelectronic nanodevices as well as high-temperature, high-power and high-frequency electronic devices [1,2,3,4]. Among the Group-III nitride semiconductors, GaN is known as one of the most promising materials for third-generation semiconductors because of its excellent properties such as a wide direct band gap, low dielectric constant, corrosion resistance, radiation resistance, high luminous efficiency, high temperature resistance and high strength [5,6,7,8,9]. In addition, GaN can be made magnetic by introducing Mn magnetic dopants and is considered to be suitable for future spintronic devices [10,11,12,13]. 

Under ambient conditions, GaN presents a hexagonal wurtzite structure, while thin films of single-crystal zinc-blende GaN have been reported to be grown on *β*-SiC (100) substrates in a modified molecular-beam epitaxy system [14]. Zinc-blende is hoped to be more amenable to doping than the wurtzite GaN [15,16]. Most of the previous studies focused on the effect of surface adsorption [17,18,19,20], defects [21,22,23] and doping [24,25,26,27] on the chemical adsorption performance and their effect on electronic and optoelectronic properties of wurtzite GaN. Li et al. [28] pointed out that the density of states around the Fermi level for the wurtzite structure are much lower than that for the zinc-blende structure, which results in a wider band gap. Luo et al. [29] investigated the electronic structure and the elastic and thermal properties of zinc-blende GaN by first-principles calculations. Thus far, for the limitation of experimental methods, systematic and in-depth studies on the mechanical and thermodynamic of wurtzite and zinc-blende GaN crystals remain very limited, especially for the anisotropic behavior of mechanical properties. Fortunately, with the rapid development of numerical methods, calculations and predictions on many mechanical and physical properties of compounds and alloys based on density functional theory (DFT) have been successfully performed and their validity has been verified [30,31,32,33].

To help understand the physical properties of the wurtzite and zinc-blende GaN crystals, establish finite element models, analyze microelectronics reliability, and design components and devices, in this study, first-principles calculations based on DFT have been applied to investigate the elastic constants, elastic properties, elastic anisotropies, thermodynamic properties and electronic properties of wurtzite and zinc-blende GaN crystals. 

## 2. Computational Method and Details

**Figure 1:**
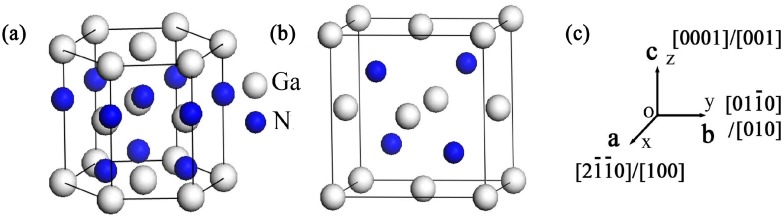
Crystal structures of: Wurtzite GaN (**a**); and zinc-blende GaN (**b**); and the relationship between *a*, *b* and *c* axes and crystallographic directions (**c**).

Under ambient conditions, GaN crystallizes in a hexagonal wurtzite structure with the space group *P*6_3_ *mc* having lattice constants *a* = *b*, *c* and lattice angles *α* = *β* = 90°, *γ* = 120° [28]. The zinc-blende GaN can be described as a cubic structure with *a* = *b* = *c* and *α* = *β* = *γ* = 90°, having the space group *F*-43*m* [28]. The two structures for GaN crystals are shown in Figure 1. In this study, first principle calculations based on DFT calculations were performed using the Cambridge Sequential Total Energy Package (CASTEP) program [34], which has already been employed successfully to calculate the physical properties of numerous alloys and compounds. The generalized gradient approximation (GGA) [35] of the revised Perdew-Burke-Ernzerhof formalism [36] and the local density approximation (LDA) proposed by Ceperley and Alder and parameterized by Perdew and Zunger (CA-PZ) were performed to calculate the exchange-correlation potential. Meanwhile, the Broyden Fletcher Goldfarb Shanno algorithm [27] and Vanderbilt ultra-soft pseudopotentials [37] was used to optimize the crystal models. The electronic wave functions were expanded with an energy cut-off of 520 eV for all calculations. For the Brillouin-zone integration, a 12 × 12 × 12 Monkhorst-Pack mesh [38] was selected for the calculations based on the variation in total energy of less than 1 meV atom^−1^ [30]. The self-consistent field tolerance was set at 5.0 × 10^−7^ eV atom^−1^. It was considered that the system would reach the ground state when the convergence precision of the energy, maximum force, maximum displacement and maximum stress were less than 5.0 × 10^−6^ eV, 0.01 eV Å^−1^, 5.0 × 10^−4^ Å and 0.02 GPa, respectively. Moreover, the valence-electron configurations of Ga and N were 3d^10^4s^2^4p^1^ and 2s^2^2p^3^, respectively, For the limitation of both GGA and LDA in calculating band gaps and structures, in this study, Heyd-Scuseria-Ernzerhof exchange-correlation function (HSE06 [39]) was applied to address the band gap problem of the two GaN crystals. The stiffness matrix ***C_ij_*** was obtained by linear fitting of four small strains (±0.001 and ±0.003) under nine deformation conditions [40], and elastic compliance matrix ***S_ij_*** was calculated as the inverse of ***C_ij_***, i.e., ***S_ij_*** = [***C_ij_***]^−1^.

## 3. Results and Discussion

### 3.1. Elastic Constants

The calculated lattice constants and equilibrium volumes in the ground state are listed in Table 1, where the experimental results of wurtzite and zinc-blende GaN crystals obtained from previous studies are also given for comparison. Obviously, the calculation results show good consistency with the experimental data, thus demonstrating the effectiveness of the calculations in this study. Moreover, the lattice parameters of wurtzite and zinc-blende structures are commonly overestimated by GGA while underestimated by LDA. The calculation results of the lattice constants obtained by LDA are closer to the experimental results as the maximum deviation of the lattice constant (herein, *a*, *b* and *c*) is about 1%, which is slightly less than that obtained by the GGA method.

The elastic constant is an essential property that can provide a link between the mechanical and dynamic behavior of crystals and give important information concerning the nature of the chemical bonding operating in solids [30]. In the stiffness matrix ***C_ij_*** of hexagonal crystals, there are five independent elastic constants, *C*_11_, *C*_12_, *C*_13_, *C*_33_ and *C*_44_, and the elastic constants for the hexagonal wurtzite GaN crystal should satisfy the following stability criteria [42]:

(1) $$C_{11}>0,\left(C_{11}-C_{12}\right)>0,C_{44}>0,\left(C_{11}+C_{12}\right)C_{33}>2C_{13}^{2}$$

In the stiffness of cubic zinc-blende GaN, there are three independent elastic constants, *C*_11_, *C*_44_ and *C*_12_; restrictions on the elastic constants are [42]:

(2) $$\left(C_{11}+2C_{12}\right)>0,C_{44}>0,\left(C_{11}-C_{12}\right)>0$$

The calculated elastic constants ***C_ij_*** and ***S_ij_*** for the two GaN crystals are listed in Table 2 and Table 3, respectively, where the previous experimental data of ***C_ij_*** are consistent with the results obtained in this work, thus proving the correctness of the simulation carried out in this paper. Clearly, according to the result shown in Table 2, the elastic constants of the two GaN crystals obey the above stability criteria.

### 3.2. Elastic Properties

The bulk modulus (*B*) is a parameter to measure how incompressible/resistant to compressibility the materials are. *B* is defined as the ratio of the increase in infinite small pressure to the resulting relative volume change and can be predicted from the calculated elastic constants within the Voigt–Reuss scheme [42]:

(3) $$B_{V}=\frac{1}{9}\left[C_{11}+C_{22}+C_{33}+2\left(C_{12}+C_{13}+C_{23}\right)\right]$$

(4) $$B_{R}={\left[S_{11}+S_{22}+S_{33}+2(S_{12}+S_{13}+S_{23})\right]}^{-1}$$

(5) $$G_{V}=\frac{1}{15}\left[\left(C_{11}+C_{22}+C_{33}\right)-\left(C_{12}+C_{13}+C_{23}\right)+3\left(C_{44}+C_{55}+C_{66}\right)\right]$$

(6) $$G_{R}=15{\left[4\left(S_{11}+S_{22}+S_{33}\right)-4\left(S_{12}+S_{13}+S_{23}\right)+3\left(S_{44}+S_{55}+S_{66}\right)\right]}^{-1}$$

where *B_V_*, *B_R_* and *G_V_*, *G_R_* stand for the upper and lower bounds of the bulk moduli and shear moduli of the polycrystalline aggregate, respectively. Moreover, the effective bulk modulus (*B*) and effective shear modulus (*G*) can be calculated using the Voigt-Reuss-Hill approximation [46], which are considered arithmetic means:

(7) $$B=\left(B_{V}+B_{R}\right)/2$$

(8) $$G=\left(G_{V}+G_{R}\right)/2$$

The effective elastic modulus (*E*) and Poisson’s ratio (*ν*) of the GaN polycrystalline aggregate can be expressed as follows [47]:

(9) $$E=\frac{9BG}{3B+G}$$

(10) $$\nu =\frac{3B-2G}{6B+2G}$$

The calculated *B*, *G*, *E*, *ν* and *B*/*G* for the two GaN polycrystals are listed in Table 4. Generally, the two GaN polycrystals have many common mechanical properties. The buck moduli of the two crystals are similar, while the other modulus values (i.e., *G* and *E*) for wurtzite GaN are a little larger, indicating that, compared to zinc-blende GaN, the stiffness of wurtzite GaN is slightly higher. According to the Pugh criterion, if *B*/*G* > 1.75, then ductile behavior may occur, otherwise the material behaves in a brittle mode [48]. Obviously, the ratio of *B* to *G* for hexagonal wurtzite GaN is 1.31 (GGA) or 1.61 (LDA), which is much less than 1.75, as given in Table 4, implying that wurtzite GaN may act like a brittle material. For zinc-blende GaN, the ratio is 1.59 (GGA) or 1.76 (LDA), i.e., it is close to 1.75. This implies that zinc-blende GaN might also be a brittle material while its brittleness is less than that of wurtzite GaN. Moreover, Poisson’s ratio (*ν*) is widely applied to evaluate the stability of the crystals under shear deformation, and a larger *ν* indicates that the materials would present a better plasticity. Zinc-blende GaN exhibits a better plastic behavior because of the larger *ν* (0.24 for GGA and 0.26 for LDA), while wurtzite GaN is more stable against shear stress for the smaller *ν* (0.20 for GGA and 0.24 for LDA). Furthermore, the value of *ν* implies the degree of directionality of the covalent bond. Its value is small (about 0.1) for covalent materials, while, for ionic materials, the typical value is 0.25 [49]. The calculated value of *ν* ranges from 0.20 to 0.27 for the two structures, indicating they are both ionic materials.

### 3.3. Elastic Anisotropy

The elastic anisotropy of materials has a significant effect on their physical properties, such as anisotropic deformation, crack propagation and elastic instability. In this section, the elastic anisotropy factor (*A*) is applied to explore the anisotropy degree of the two GaN monocrystals [56], as given in Table 5. If the value of anisotropy factor *A* is close to 1, then the mechanical behavior of the plane is prone to isotropy. The calculated results show that, in wurtzite GaN, *A* of planes containing the [001] axis (i.e., [0001] axis or orientation) is far from 1, indicating wurtzite GaN displays obvious anisotropic characteristics in these planes. In addition, zinc-blende GaN presents strong anisotropy in its crystal planes {100} and {110}.

To further investigate the anisotropic characteristics of monocrystal GaN crystals, three-dimensional surfaces revealing the elastic anisotropy were analyzed. The direction dependence of the elastic modulus of general monocrystals is given as follows [42]:

(11) $$\begin{array}{l} 1/E=S_{11}l_{1}^{4}+2S_{12}{\left(l_{1}l_{2}\right)}^{2}+2S_{13}{\left(l_{1}l_{3}\right)}^{2}+2S_{14}(l_{1}^{2}l_{2}l_{3})+2S_{15}(l_{3}l_{1}^{3})+2S_{16}(l_{1}^{3}l_{2})\\ \;+S_{22}l_{2}^{4}\;+2S_{23}{\left(l_{2}l_{3}\right)}^{2}+2S_{24}(l_{2}^{3}l_{3})+2S_{25}(l_{1}l_{2}^{2}l_{3})+2S_{26}(l_{1}l_{2}^{3})\\ \;+S_{33}l_{3}^{4}\;+2S_{34}(l_{2}l_{3}^{3})+2S_{35}(l_{1}l_{3}^{3})+2S_{36}(l_{1}l_{2}l_{3}^{2})\\ \;+S_{44}{\left(l_{2}l_{3}\right)}^{2}\;+2S_{45}(l_{1}l_{2}l_{3}^{2})+2S_{46}(l_{1}l_{3}l_{2}^{2})\\ \;+S_{55}{\left(l_{1}l_{3}\right)}^{2}\;+2S_{56}(l_{2}l_{3}l_{1}^{2})\\ \;+S_{66}{\left(l_{1}l_{2}\right)}^{2} \end{array}$$

where *l*_1_, *l*_2_ and *l*_3_ are the direction cosines concerning the *a*, *b* and *c* directions of the crystal lattices, respectively, and ***S_ij_*** (*i*, *j* = 1, 2, 3) are the compliance coefficients, which are listed in Table 3. According to Equation (11) and the crystal features of hexagonal and cubic monocrystals, the direction dependences of the elastic moduli of the wurtzite and zinc-blende can be expressed as in Equations (12) and (13), respectively.

(12) $$\frac{1}{E}={\left(1-l_{3}^{2}\right)}^{2}S_{11}+l_{3}^{4}S_{33}+l_{3}^{2}(1-l_{3}^{2})(2S_{13}+S_{44})$$

(13) $$\frac{1}{E}=S_{11}+2(S_{11}-S_{12}-0.5S_{44})(l_{1}^{2}l_{2}^{2}+l_{2}^{2}l_{3}^{2}+l_{3}^{2}l_{1}^{2})$$

**Figure 2:**
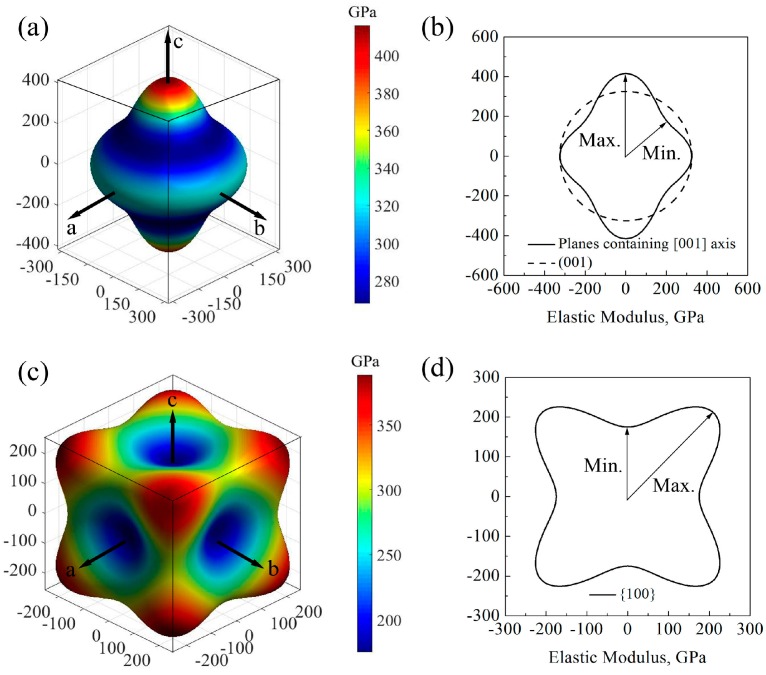
Direction dependences of elastic moduli: (**a**) wurtzite GaN; (**b**) projections on main crystal planes of wurtzite GaN, where the solid line denotes planes containing [001] axis and the dash line is plane [001]; (**c**) zinc-blende GaN; and (**d**) the projection on planes {111} of zinc-blende GaN.

The simulation results of direction dependences of the elastic moduli calculated by GGA and LDA are similar, and LDA method was applied for simulating the direction dependences of the elastic moduli for the higher accuracy in calculating structural parameters and elastic constants. Figure 2 shows the direction dependences of the elastic moduli and their projections on the main planes of GaN crystals. For wurtzite GaN, the shape of elastic modulus at plane (001) is a circle and the value is 324.8 GPa, while it is far from a circle at planes containing [001] axis (e.g., planes ($01\bar{1}0$) and ($2\bar{1}\bar{1}0$)) (Figure 2a,b); therefore, the elastic modulus of wurtzite GaN may exhibit an isotropic characteristic at the plane (001) while it shows significant anisotropy at the planes containing [001] axis, where the maximum and minimum values are 415.8 GPa and 267.8 GPa, respectively. For zinc-blende GaN, the elastic moduli along the [100] axis (i.e., *E_x_*), [010] axis (i.e., *E_y_*) and the [001] axis (i.e., *E_z_*) are all the minimum value 175.0 GPa, and the maximum elastic moduli are located at the orientations <111> and the value is 388.5 GPa, as shown in Figure 2c. In addition, in the zinc-blende GaN, there are obvious anisotropic characteristics for all small miller indices crystallographic planes, and Figure 2d shows that the maximum and minimum elastic moduli in the projection of planes {100} are 297.7 and 175.0 GPa, respectively.

**Figure 3:**
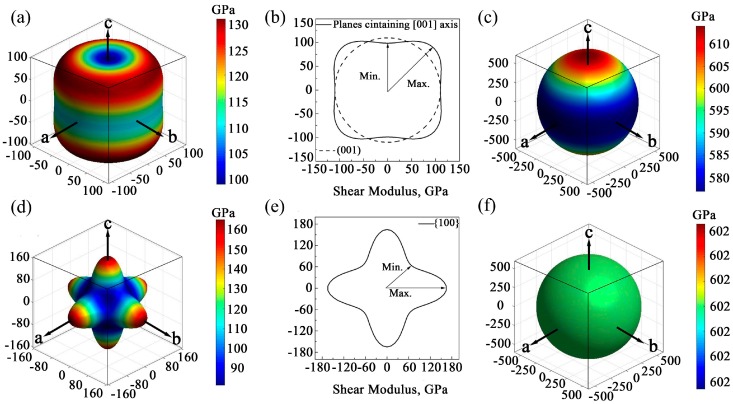
Directional dependence of elastic properties: (**a**) shear modulus of wurtzite GaN; (**b**) the projection of shear modulus of planes containing [001] axis in the wurtzite GaN; (**c**) bulk modulus of wurtzite GaN; (**d**) shear modulus of zinc-blende GaN; (**e**) the projection of shear modulus of planes {100} in the zinc-blende GaN; and (**f**) bulk modulus of zinc-blende GaN.

Moreover, shear moduli of hexagonal wurtzite GaN and cubic zinc-blende GaN can be expressed as Equation (14) [57] and Equations (15)–(17), respectively,

(14) $$\frac{1}{G}=S_{55}+\left(S_{11}-S_{12}-\frac{S_{44}}{2}\right)\left(1-l_{3}^{2}\right)+2\left(S_{11}+S_{33}-2S_{13}-S_{44}\right)\left(1-l_{3}^{2}\right)l_{3}^{2}$$

(15) $$\frac{1}{G}=\left(S_{44}+4S_{0}J\right)$$

(16) $$S_{0}=S_{11}-S_{12}-\frac{1}{2}S_{44}$$

(17) $$J=\sin^{2}\theta \cdot \cos^{2}\theta +0.125\cdot \sin^{4}\theta \left(1-\cos4\phi \right)$$

where *θ* and *φ* are the Euler angles in the measurement systems. Figure 3a,b,d shows the direction dependences of the shear moduli and their projections on the main planes of GaN crystals. For wurtzite GaN, it is found that the shape of directional shear modulus at the (001) plane is a circle, while it is far from the circle at the planes containing the [001] axis. Therefore, the shear modulus of wurtzite GaN may exhibit an isotropic characteristic at the plane (001) and the isotropic value is 110.0 GPa, while it shows significant anisotropy at planes containing [001] axis, and the maximum and minimum values are 130.8 GPa and 98.9 GPa, respectively (see Figure 3a,b). For zinc-blende GaN, the shear moduli along the [100] axis (i.e., *E_x_*), [010] axis (i.e., *E_y_*) and the [001] axis (i.e., *E_z_*) are all the maximum value 165.0 GPa, and the minimum shear moduli are located at the orientations <111> and the value is 81.0 GPa (see Figure 3d). In addition, in the zinc-blende GaN, there are also obvious anisotropic characteristics for all small miller indices crystallographic planes, and Figure 3e shows that the maximum and minimum shear moduli in the projection of planes {100} are 165.0 and 92.8 GPa, respectively. Obviously, to some extent, the shear modulus of GaN monocrystals has an inverse direction dependence compared to the elastic modulus. In summary, the elastic moduli and shear moduli of GaN monocrystals exhibit obvious anisotropy behavior, while the following calculation shows that the bulk moduli of the two monocrystals are nearly isotropic. Directional dependences of bulk moduli of hexagonal and cubic GaN monocrystal can be calculated using Equations (18) and (19), respectively,

(18) $$\frac{1}{B}=(S_{11}+S_{12}+S_{13})-(S_{11}+S_{12}-S_{13}-S_{33})l_{3}^{2}$$

(19) $$\frac{1}{B}=\left(S_{11}+2S_{12}\right)\left(l_{1}^{2}+l_{2}^{2}+l_{3}^{2}\right)$$

For wurtzite GaN, the maximum bulk modulus is located at crystal orientations which are vertical to [001] ([0001]) axis, e.g., [100] ($2\bar{1}\bar{1}0$), [010] ($\left[01\bar{1}0\right]$), etc., and its value is 614.0 GPa. Besides, the minimum bulk modulus is in the direction of [100] axis and the value is 576.8 GPa, as shown in Figure 3c. Obviously, there is very slight anisotropy in the wurtzite GaN. For zinc-blende GaN, there is no anisotropy of bulk modulus, and the value is 602.4 GPa in all orientations (see Figure 3f). 

Further, the curves of Poisson’s ratios at the planes containing [001] axis *v*(*θ*) in the wurtzite GaN can be expressed as Equation (20) [58], and Poisson’s ratios at the plane (*hkl*) of zinc-blende GaN can be described as Equation (21) [59],

(20) $$v(\theta )=\frac{s_{12}\sin^{2}\theta +s_{13}\cos^{2}\theta }{s_{11}\sin^{4}\theta +s_{33}\cos^{4}\theta +(s_{44}+2s_{13})\sin^{2}\theta \cos^{2}\theta }$$

(21) $$\begin{array}{l} \nu \left(hkl,\theta \right)=\{S_{12}+\frac{S_{0}}{h^{2}+k^{2}+l^{2}}{[\left(\frac{h^{2}l}{\sqrt{h^{2}+k^{2}}\sqrt{h^{2}+k^{2}+l^{2}}}\cos\theta -\frac{hk}{\sqrt{h^{2}+k^{2}}}\sin\theta \right)}^{2}+\\ (\frac{k^{2}l}{\sqrt{h^{2}+k^{2}}\sqrt{h^{2}+k^{2}+l^{2}}}{\times \cos\theta +\frac{hk}{\sqrt{h^{2}+k^{2}}}\sin\theta )}^{2}+{\left(\frac{l\sqrt{h^{2}+k^{2}}}{\sqrt{h^{2}+k^{2}+l^{2}}}\cos\theta \right)}^{2}]\}\\ /\left[-S_{11}+2S_{0}\frac{{\left(hk\right)}^{2}+{\left(hl\right)}^{2}+{\left(lk\right)}^{2}}{{\left(h^{2}+k^{2}+l^{2}\right)}^{2}}\right] \end{array}$$

**Figure 4:**
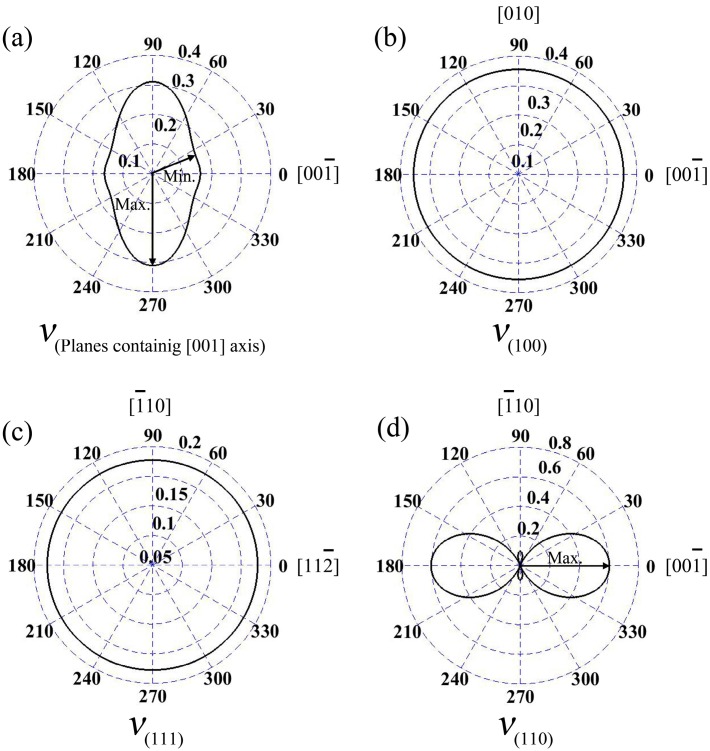
Poisson’s ratios *v* for the planes containing [001] ([0001]) axis in the wurtzite GaN (**a**); and three low index planes (100) (**b**); (111) (**c**); and (110) (**d**) in the zinc-blende GaN.

In the wurtzite GaN, Poisson’s ratios at the planes containing [001] axis are illustrated in Figure 4a; the maximum value is 0.31, and it is located at the directions vertical to [001] axis. Meanwhile, the minimum *ν* is 0.158, and the angle between the direction and [001] axis is 19.8°. In addition, *ν* along [001] axis of wurtzite GaN is 0.162. Additionally, for zinc-blende GaN, Poisson’s ratios at planes (100) and (111) are isotropic, as shown in Figure 4b,c, and the values are 0.35 and 0.18, respectively. However, *ν* at plane (110) exhibits obvious anisotropic behavior (see Figure 4d), where the maximum value 0.60 is located at [001] and $\left[00\bar{1}\right]$ directions, while the minimum value is 0.10 (actually, −0.10) and it is in the orientations $\left[\bar{1}10\right]$ or $\left[1\bar{1}0\right]$.

### 3.4.Thermodynamic Properties

The calculation results of phonon spectra can be used to compute lattice heat capacity (*C_V_*) as functions of temperature [60]. The temperature dependence of the energy can be calculated as follows:

(22) $$E\left(T\right)=E_{tot}+E_{zp}+\int \frac{\hbar \omega }{\exp\left(\frac{\hbar \omega }{kT}\right)-1}F\left(\omega \right)d\omega$$

where *E_tot_* is the total static energy at 0 K, which can be calculated by first principles, *E_zp_* is the zero-point vibrational energy, *ω* is the frequency, *k* is the Boltzmann’s constant, *T* is the Kelvin temperature, *ħ* is Planck’s constant and *F*(*ω*) is the phonon density of states. *E_zp_* can be expressed as follows:

(23) $$E_{zp}=\frac{1}{2}\int F\left(\omega \right)\hbar \omega d\omega$$

The lattice contribution to the heat capacity, *C_V_*, is calculated as follows:

(24) $$C_{V}\left(t\right)=k\int \frac{{\left(\frac{\hbar \omega }{kT}\right)}^{2}\exp\left(\frac{\hbar \omega }{kT}\right)}{{\left[\exp\left(\frac{\hbar \omega }{kT}\right)-1\right]}^{2}}F\left(\omega \right)d\omega$$

**Figure 5:**
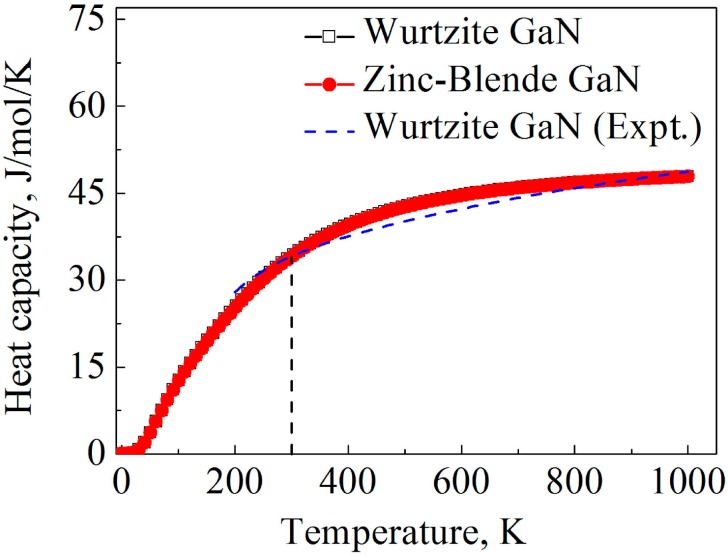
Calculation results of temperature-dependent heat capacities for wurtzite and zinc-blende GaN. The dash line denotes experimental data of wurtzite GaN obtained from Ref. [62].

The heat capacity calculated by the LDA method is illustrated in Figure 5. At low temperatures, power-law temperature dependence is clearly observed for the heat capacity, and the predicted power-law exponent is approximately 3 for the two GaN crystals according to the calculation result, which is consistent with the Debye heat capacity theory. Moreover, at 300 K, the calculated heat capacities of wurtzite and zinc-blende are 34.4 and 34.3 J mol^−1^ K^−1^, respectively, which are close to the experimental value of wurtzite GaN at 300 K (34.1 J mol^−1^ K^−1^) [61]. At high temperatures, the heat capacity of the two GaN crystals tends to converge at 49.4 mol^−1^ K^−1^, which is the Dulong–Petit limit. Obviously, heat capacities of the two GaN crystals are quite close as the temperature is increased from 0 to 1000 K, and the effect of crystal structures on the heat capacity of GaN is very limited. 

Further, Debye temperature is one of important basic physical parameters, which has connections with many mechanical and physical properties such as elastic constants, heat capacity, bond strength, and so on [62]. By employing elastic constants and average sound velocity *ν_m_*, Debye temperature Θ*_D_* can be approximately calculated by

(25) $$\Theta_{D}=\frac{\hbar }{k}{\left(\frac{3nN_{A}\rho }{4\pi M}\right)}^{1/3}v_{m}$$

(26) $$v_{m}={\left[\frac{1}{3}\left(\frac{1}{v_{l}^{3}}+\frac{2}{v_{t}^{3}}\right)\right]}^{-1/3}$$

where *n* is the number of atoms in the cell, *N*_A_ is Avogadro constant, *M* is the molecular weight, *ρ* is the density, and *ν_t_* and *ν_l_* are longitudinal and transverse sound velocities, respectively, which can be obtained from Equations (27) and (28), respectively.

(27) $$v_{t}={\left(\frac{G}{\rho }\right)}^{1/2}$$

(28) $$v_{l}={\left(\frac{3B+4G}{3\rho }\right)}^{1/2}$$

The thermodynamic properties of GaN crystals calculated by LDA method are listed in Table 6. Obviously, the densities of the two structure GaN crystals are extremely close, and wurtzite GaN has higher *ν_t_*, *ν_l_* and *ν_m_*. Besides, the measured Debye temperatures of GaN are scattered, and previous study reported that the experimental measured Debye temperatures of wurtzite GaN range from 586 K to 898 K [63]. In this study, the calculated Debye temperatures of wurtzite and zinc-blende GaN are 641.8 and 620.2 K, respectively. Compared with zinc-blende GaN, wurtzite GaN has the slightly higher Debye temperature, indicating stronger Ga–N ionic bonds in the wurtzite GaN. Therefore, wurtzite GaN exhibits slightly higher stiffness and melt point to a small degree.

### 3.5. Electronic Properties

**Figure 6:**
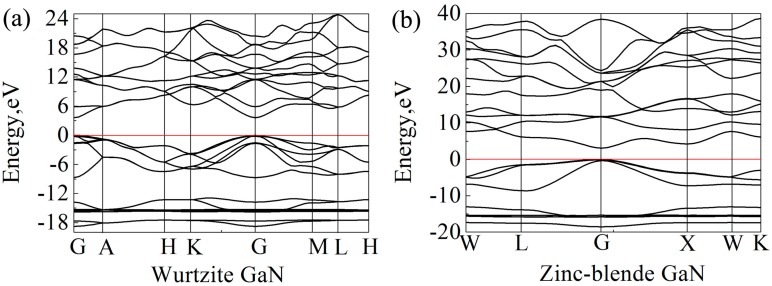
Band structures of GaN crystals: (**a**) Wurtzite GaN; and (**b**) zinc-blende GaN. The Fermi level is set to zero (see the red line).

**Figure 7:**
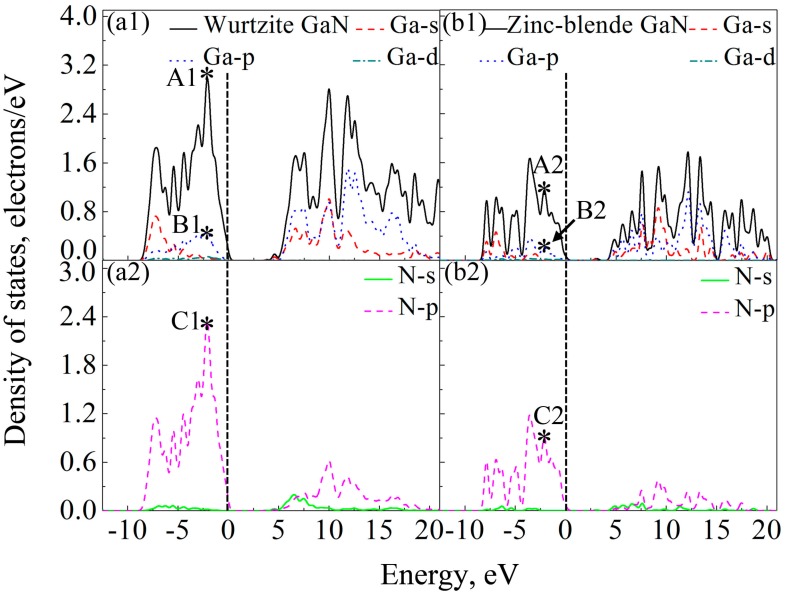
DOSs for the two GaN crystals and their atoms: (**a1**) wurtzite GaN and Ga atoms; (**a2**) N atoms in wurtzite GaN; (**b1**) zinc-blende GaN and Ga atoms; and (**b2**) N atoms in zinc-blende GaN. The mark * indicates the peak of DOS at −2.1 eV

Band gap is the energy required for a valence electron to become a conduction electron, which moves freely and serves as a charge carrier. According to previous experimental results, the band gaps of wurtzite and zinc-blende GaN crystals are 3.5 eV [55] and 3.1 eV [64], respectively. However, Band gaps of wurtzite and zinc-blende GaN crystals calculated by LDA are 1.66 eV and 1.48 eV, respectively, and they are 2.01 and 1.91 eV by GGA. Here, HSE06 scheme [39] was applied for band gap calculations, and the calculated band gaps of wurtzite and zinc-blende GaN are 3.62 eV and 3.06 eV, respectively, which are close to the experimental values. Clearly, the results also show that the band gap of wurtzite GaN is larger than that of zinc-blende GaN. In previous studies, it is reported that the band gap of wurtzite GaN is located at the Γ (G) point [65,66]. In the present study, the band structures of wurtzite and zinc-blende GaN crystals are shown in Figure 6. It is also found that the maximum value of valence band and the minimum value of conduction band are located at the G point for the two crystals, indicating that all of them are direct gap semiconductors. At the G point, the lowest energy of conduction band in the wurtzite GaN is larger than that in the zinc-blende GaN, which results in a wider band gap for the wurtzite GaN. Figure 7 shows DOSs of the GaN crystals around band gaps. According to the distribution characteristics of DOSs of GaN crystals, the two crystals exhibit similar atomic bonding and hybrid behavior. In the energy band with the energy ranging from −10 to 0 eV, DOSs of the two GaN crystals are primarily contributed by p orbits of N atoms (N-p). However, for the energy band in the conduction band, which is close to the band gap, e.g., the energies ranging from 3.62 eV to 20 eV for wurtzite GaN, DOSs of GaN crystals are mainly due to the hybridization between N-p and s and p orbits of Ga (Ga-s and Ga-p). Meanwhile, although the two crystals exhibit similar atomic bonding and hybridization behavior, the DOSs of Ga atoms, N atoms and wurtzite GaN are much larger than those of zinc-blende GaN, as observed in Figure 7. For example, the values of A1 (peak of DOS of GaN), B1 (peak of Ga-p) and C1 (peak of N-p) at −2.1 eV are 2.98, 0.48 and 2.36 electrons/eV, respectively, while A2, B2 and C2 at −2.1 eV for zinc-blende GaN are 1.15, 0.22 and 0.91 electrons/eV, respectively. Obviously, the values of A1, B1 and C1 are much larger than those of A2, B2 and C2, respectively. Therefore, it can be inferred that more electrons in corresponding atomic orbitals participate in forming Ga-N ionic bonds in the wurtzite GaN, which is why the wurtzite GaN has larger elastic modulus, higher Debye temperature, smaller Poisson’s ratio and shows more evident characteristic of brittleness.

## 4. Conclusions

The mechanical, thermodynamic and electronic properties of wurtzite and zinc-Blende GaN crystals were investigated by first-principles calculations based on density functional theory. From the present study, the following conclusions can be drawn:

1. Generally, the two GaN polycrystals have common or similar elastic properties. The bulk moduli of the wurtzite and zinc-blende GaN polycrystals are very close, and polycrystalline wurtzite GaN has slightly larger shear modulus and elastic modulus but smaller Poisson’s ratio and shows more evident characteristic of brittleness.
2. Except for the bulk modulus, the anisotropic behavior of the two GaN monocrystal is quite different, or even opposite. For wurtzite GaN monocrystal, the maximum and minimum elastic moduli are located at orientations [001] and <111>, respectively, while they are in the orientations <111> and <100> in the monocrystal zinc-blende GaN, respectively. Compared to their respective elastic moduli, the shear moduli of the two GaN monocrystals have completely opposite direction dependences. Thus, the differences in their anisotropic behavior should draw enough attention.
3. For wurtzite GaN, Poisson’s ratios at the planes containing [001] axis are anisotropic. The maximum value is 0.31 and it is located at the directions vertical to [001] axis. For zinc-blende GaN, Poisson’s ratios at planes (100) and (111) are isotropic, while the Poisson’s ratio at plane (110) exhibits dramatically anisotropic phenomenon.
4. The Debye temperatures calculated based on elastic constants and average sound velocities of wurtzite and zinc-blende GaN are 641.8 and 620.2 K, respectively, and wurtzite GaN exhibits slightly higher stiffness and melt point for the higher Debye temperature. At the same temperature, the two GaN crystals have the same heat capacity. Zinc-blende GaN is hoped to be more amenable to doping than the wurtzite GaN [15,16], thus it can be a first decent approach of wurtzite GaN due to many common or similar properties.
5. The exchange-correlation functional theory HSE06 is recommended to calculate the band gaps. Band gaps are located at the G point for the two crystals, and the band gaps of wurtzite and zinc-blende GaN are 3.62 and 3.06 eV, respectively. At the G point, the lowest energy of conduction band in the wurtzite GaN is larger than that in the zinc-blende GaN, resulting in a wider band gap.
6. Densities of states in the orbital hybridization between Ga and N atoms of wurtzite GaN are much higher, indicating more electrons in corresponding atomic orbitals participate in forming Ga-N ionic bonds, which is why the wurtzite GaN has larger elastic modulus, higher Debye temperature, smaller Poisson’s ratio and shows more evident characteristic of brittleness.

## Figures and Tables

**Table 1:** Lattice constants of GaN crystals.

Structure	Method	Lattice Constants			
		*a*_0_ = *b*_0_ (Å)	*c*_0_ (Å)	*c*_0_/*a*_0_	*V*_0_
Wurtzite GaN	GGA (present)	3.242	5.280	1.629	48.075
	LDA (present)	3.156	5.145	1.631	44.373
	Expt. [41]	3.189	5.185	1.626	45.671
Zinc-blende GaN	GGA (present)	4.582	4.582	1.000	96.221
	LDA (present)	4.461	4.461	1.000	88.800
	Expt. [15]	4.490	4.490	1.000	90.519

**Table 2:** Calculated elastic constant ***C_ij_***, GPa.

Structure	*C*_11_ = *C*_22_	*C*_12_	*C*_13_ = *C*_23_	*C*_33_	*C*_44_ = *C*_55_	*C*_66_	RMSE *	Note
Wurtzite GaN	353.93	82.59	51.79	380.44	98.49	135.67	37.90	GGA
	374.35	126.56	80.85	441.94	98.86	123.89	21.35	LDA
	390 ± 15	145 ± 20	106 ± 20	398 ± 20	105 ± 10	123 ± 10	**-**	Expt. [43]
Zinc-blende GaN	242.41	122.05	122.05	242.41	146.82	146.82	**-**	GGA
	286.93	152.77	152.77	286.93	164.97	164.97	**-**	LDA
	285	161	161	285	149	149	**-**	LDA [44]
	293	159	159	293	155	155	**-**	LDA [45]

* Root Mean Square Error.

**Table 3:** Calculated elastic constant ***S_ij_***, × 10^−3^/GPa.

Structure	*S*_11_*=S*_22_	*S*_12_	*S*_13_*=* *S*_23_	*S*_33_	*S*_44_	*S*_55_	*S*_66_	Note
Wurtzite GaN	3.026	−0.659	−0.322	2.716	10.153	10.153	7.371	GGA
	3.079	−0.957	−0.388	2.405	10.115	10.115	8.071	LDA
Zinc-blende GaN	6.224	−2.084	−2.084	6.224	6.811	6.811	6.811	GGA
	5.712	−2.027	−2.027	5.712	6.061	6.061	6.061	LDA

**Table 4:** Bulk modulus *B*, shear modulus *G*, elastic modulus *E*, Poisson’s ratio *ν* and *B*/*G* of polycrystalline GaN.

Structure	*B*, GPa	*G*, GPa	*E*, GPa	*ν*	*B/G*	Note
Wurtzite GaN	162.3	124.1	296.7	0.20	1.31	GGA
	196.3	121.7	302.6	0.24	1.61	LDA
	170 [50] 188 [51]	116 [52]	295 ± 3 [53]	0.23 ± 0.06 [54]	-	Expt.
Zinc-blende GaN	162.2	102.2	253.4	0.24	1.59	GGA
	199.1	113.1	285.3	0.26	1.76	LDA
	203.7	110.71	281.18	0.27	1.84	Expt. [55]

**Table 5:** Anisotropy factor *A* [56].

Structure	Symmetry	Anisotropy Factor *A*	Calculation Results	
			GGA	LDA
Wurtzite GaN	Planes containing the [001] axis	$C_{44}\left(C_{11}+2C_{13}+C_{33}\right)/\left(C_{11}C_{33}-C_{13}^{2}\right)$	0.63	0.61
Zinc-blende GaN	{100}	$2C_{44}/\left(C_{11}-C_{12}\right)$	2.44	2.46
	{110}	$C_{44}\left(C_{L}+2C_{12}+C_{11}\right)/(C_{L}C_{11}-C_{12}^{2})$*	1.85	1.85

* For cubic crystals *C_L_* = *C*_66_ + (*C*_11_ + *C*_12_)/2.

**Table 6:** The calculated thermodynamic properties of GaN.

Structure	*ρ*, g/cm	*ν_t_*, m/s	*ν_l_*, m/s	*ν_m_*, m/s	*θ_D_*, K
Wurtzite GaN	6.268	4406.386	7563.581	4888.006	641.8
Zinc-blende GaN	6.264	4249.685	7474.802	4724.200	620.2
